# Mouthwash Containing Vitamin E, Triamcinolon, and Hyaluronic Acid Compared to Triamcinolone Mouthwash Alone in Patients With Radiotherapy-Induced Oral Mucositis: Randomized Clinical Trial

**DOI:** 10.3389/fonc.2021.614877

**Published:** 2021-03-11

**Authors:** Farzaneh Agha-Hosseini, Mona Pourpasha, Massoud Amanlou, Mahdieh-Sadat Moosavi

**Affiliations:** ^1^ Dental Research Center, Dentistry Research Institute, Tehran University of Medical Sciences, Tehran, Iran; ^2^ Department of Oral Medicine, Faculty of Dentistry, Tehran University of Medical Sciences, Tehran, Iran; ^3^ The Academy of Medical Sciences Tehran, Tehran, Iran; ^4^ Department of Medicinal Chemistry, Faculty of Pharmacy, Tehran University of Medical Sciences, Tehran, Iran

**Keywords:** head and neck cancer, hyaluronic acid, mucositis, radiotherapy, vitamin E

## Abstract

One of the most common side effects of radiotherapy in head and neck cancers is mucositis. Despite all the studies conducted on new therapies proposed for oral mucositis caused by radiation therapy, a single standard treatment strategy has not been developed yet. In the present study, for the first time, the effectiveness of the treatment with a combined mouthwash containing vitamin E (as an antioxidant), triamcinolone (as an anti-inflammatory agent) and hyaluronic acid (HA) (as a local reducer used for reducing the effects of ROS on the mucosa, with ameliorative effects (improving the healing process) compared to triamcinolone mouthwash alone was investigated in patients with radiotherapy-induced oral mucositis. This study was a randomized triple-blind clinical trial performed on 60 patients underwent radiotherapy on an outpatient basis. The combined mouthwash containing vitamin E, triamcinolone, and hyaluronic acid compared to triamcinolone mouthwash alone was prescribed for 4 weeks. The severity of oral mucositis was assessed based on the WHO classification and the intensity of pain was assessed using the numerical pain intensity scale. According to the analysis performed in the first, second, third and fourth weeks, the reduction of oral mucositis grade in the intervention group was significantly higher than in the comparison group. In the first, second, third, and fourth weeks, the reduction in pain intensity in the intervention group was significantly higher than in the comparison group (P < 0.001). The combined mouthwash containing vitamin E, hyaluronic acid and triamcinolone acetonide can be used as an effective treatment for oral mucositis caused by radiation therapy, which is probably the result of antioxidant, anti-inflammatory and improved healing process mechanisms due to the biological nature of the components of this mouthwash.

**Trial registration:**

This study was registered in the WHO Primary registry (IRCT) with the code IRCT20190428043407N. Registered on 20 July 2019, https://www.irct.ir/trial/39231.

## Introduction

One of the most common side effects of radiotherapy in head and neck cancers is mucositis (1). Studies have shown that 73–100% of patients treated with intensity-modulated radiation therapy (IMRT) experienced grade 3 and 4 mucositis (2). Finding a cure for mucositis is important in several ways. Oral mucositis is often painful and manifests as erythema or sores in the oral mucosa and can also affect the mucous membranes of the throat, larynx, and esophagus (3). Therefore, it can be said that mucositis significantly affects nutrition, oral care, and quality of life. In severe cases, mucositis can lead to the need of reducing the dose of chemotherapy and undesirable breaks in radiation therapy, which would consequently have a negative effect on treatment prognosis (4).

Several mechanisms have been identified in the etiology of oral mucositis. One of them is the Nuclear Factor NF-κB signal transduction pathway. In oral mucositis, NF-κB is involved in the initial inflammatory damage to connective tissue by increasing the expression of pro-inflammatory cytokines, including TNF-a, MMPs, COX-2, TGF-B, and IL-1B (5). Another mechanism is the production of free radicals during radiation and the generation of oxidative stress (6).

Despite all the studies conducted on new therapies for oral mucositis caused by radiation therapy, a single standard treatment strategy has not yet been developed, and studies carried out on the prevention and/or treatment of oral mucositis compared with the control group have been accompanied by contradictory results and have not been fully confirmed (7). Each of these studies has introduced some materials or biomaterials in this field to block the pathway involved in the development of mucositis.

Due to the production of free radicals during radiation, several efforts have been made to use vitamin E ointment as an antioxidant and free radical scavenger (8). The main ingredient in vitamin E, alpha-tocopherol is an antioxidant that can react with free radicals and remove them from the body and better control inflammation caused by oral mucositis. Previous studies conducted on vitamin E as a radioprotective agent have reported good results (9).

Hyaluronic acid (HA) is a natural polysaccharide with a linear chain, which is known as an important element of the extracellular matrix of many tissues in the human body (10). Hyaluronic acid, while creating a protective physical barrier (coating), repairs tissues and promotes cell proliferation by regulating inflammatory responses, and besides stimulating the proliferation of basal layer keratinocytes, gives rise to re-epithelialization, improves the healing process and plays a role in reducing the size of the erosive/injured areas of the oral mucosa (11).

Due to the pathogenesis of mucositis, corticosteroid compounds such as triamcinolone acetonide are among the common treatments for this lesion. Triamcinolone is effective in reducing pain and the course of oral mucositis caused by radiation therapy. By reducing the expression of the NFKB/P65 gene and protein, these compounds reduce the levels of anti-inflammatory cytokines such as TNF-a and IL-6, and by the same mechanism, they can reduce ulcers and inflammation in oral mucositis (12).

In the present study, for the first time, the effectiveness of treatment with a combined mouthwash containing vitamin E (as an antioxidant), triamcinolone (as an anti-inflammatory agent) and hyaluronic acid (HA) (as a local reducer used for reducing the effects of ROS on the mucosa, with ameliorative effects (improving the healing process) compared to triamcinolone mouthwash alone was investigated in patients with radiotherapy-induced oral mucositis.

## Materials and Methods

This study was a randomized triple-blind clinical trial registered in the WHO Primary registry with the code (IRCT20190428043407N1) and approved by the ethics committee with the code (IR.TUMS.DENTISTRY.REC.1398.043). This study was conducted in parallel design to compare the effectiveness of a combined mouthwash containing vitamin E, hyaluronic acid and triamcinolone with triamcinolone mouthwash alone for the treatment of oral mucositis (grades 3 and 4) caused by radiotherapy. The patients entered the study by signing an informed consent form. Their ratio in the two study groups was one to one.

The study population included patients with any type of head and neck malignancy undergoing radiotherapy (IMRT) on an outpatient basis who referred to the university’s cancer institute. The patients were treated by radiotherapy up to a total dose of 60–66 Gy during 30–33 treatment sessions. Most of the patients included in this study were in the range of 10th–25th sessions of radiation therapy. The average number of previous sessions of the treatment before the start of the study was 18.57 for the intervention group and 18.58 for the control group. This study aimed to investigate the therapeutic effect of mouthwash on grades 3 and 4 of oral mucositis, so the dose delivered to the oral cavity as an OAR (Organ at Risk) was not evaluated as an independent parameter.

After the start of radiotherapy, the patients’ oral mucosa was examined every week for the incidence of oral mucositis by a specialist in oral and maxillofacial diseases.

The inclusion criteria included:

- The definitive diagnosis of head and neck cancer according to a histopathological examination and undergoing radiotherapy
- Aged at least 18 years old (no maximum age limit)
- Observing oral hygiene in a way that does not preclude the diagnosis of the degree of severity of mucositis.
- Having the ability to use mouthwash
- Patients with grades 3 and 4 of oral mucositis according to the WHO criteria, were selected for the study
- Having no history of allergy to the drugs studied (questioning patients before entering the study)
- Not having undergone other selected treatments for oral mucositis

The exclusion criteria included pregnant women, patients’ use of vitamin E and other complementary antioxidants in the last 3 weeks, suffering from other active lesions in their mouth like pests, history of alcohol or drug use, history of any previous radiation therapy and current chemotherapy (for better matching the case and control groups in terms of the type of treatment and the presence or the absence of chemotherapy affects the tissue response to treatment), and bone marrow transplantation. Additionally, systemic diseases affecting the healing process of mucosal wounds (such as diabetes and hypertension) or with oral complications (such as kidney disease and autoimmune disorders) were excluded.

The Karnofsky performance status scale below 60 is known as an index used to evaluate dysfunction, as that the smaller the number, the lower the patient’s survival rate. Moreover, some patients with an index below 60 require hospitalization.

The drug was prepared in the Medicinal Chemistry Laboratory of Tehran University of Medical Sciences by a Pharmacist according to the above-mentioned method. Thereafter, the drugs were prepared in similar bottles and then coded by a person who was not involved in the study.

The random allocation method was based on the simple randomization method using Microsoft Excel software and the randomization was performed by a person who was independent of the study.

To provide the same conditions for all the patients, candidiasis was monitored to be treated if necessary. Oral hygiene was also recommended for all the patients.

In this study, researchers, patients, and statistical analyzers did not know whom patients were in the intervention group and whom were in the control group. Enrolments of the subjects are shown in the CONSORT diagram.

### Intervention Group

The combined mouthwash was prepared with 0.1% triamcinolone (13), 0.2% vitamin E (14), and 0.2% hyaluronic acid (15). Triamcinolone acetonide powder along with vitamin E and hyaluronic acid (with the mentioned percentages) were dissolved in propylene glycol solvent and were then brought to the required volume with double-distilled water and packed in identical glass containers unrecognizable from the glass containers of the comparison group with the dropper.

### Comparison Group

0.1% triamcinolone mouthwash alone:

0.1% Triamcinolone (13) was dissolved in propylene glycol solvent, was brought to the required volume with double-distilled water, and then packed in identical glass containers that were unrecognizable from the glass containers of the intervention group with the dropper by a person who was not involved in the study, so that the drugs used for the intervention and comparison groups were similar in terms of volume and shape.

In both intervention and comparison groups, mouthwash was used for 4 weeks (16, 17), three times a day (morning, noon and night) at a rate of 2 ccs each time (without the need for dilution) and for at least one minute, without swallowing any mouthwash. It was recommended eating and drinking be avoided for 15 minutes after using the mouthwash.

During the use of mouthwash, any symptoms that indicated an allergy, such as swelling of the lips, tongue, and eyelids, hives and itching in the body gave us the permission to interrupt the intervention.

The following actions were performed during the follow-up study of patients to ensure the correct use of the drug: asking the patient’s companion about regular use of mouthwash, learning how to use mouthwash properly, patients’ weekly follow-up schedule, checking the amount of medicine left over in the glass bottle in the last week, and making phone calls.

Our patients’ cooperation in using the patient’s diaries seemed unlikely due to their illness. Therefore, as mentioned earlier, some other ways such as telephone call to the patient during the treatment period and the amount of leftover medicine were used.

### Outcome Measurements

#### The Severity of Oral Mucositis

The severity of oral mucositis was determined based on the WHO classification (13). After beginning the intervention, this variable was evaluated in the first, second, third and fourth weeks. The grade of mucositis was determined by the same oncologist. The scale was then re-examined by the same oral medicine specialist who has been trained in this field.

#### Pain Intensity

Pain intensity was assessed using the numerical pain intensity scale [a segmented numeric version of the visual analog scale (VAS)]. In this regard, a score of 10 is allocated to indescribable and severe pain and a score of 0 is for the painless state. Thus, the patient’s oral condition, including the grade of oral mucositis and pain intensity, and baseline were recorded in the first, second, third, and fourth weeks as well as weak zero, respectively.

### Statistical Analysis

The outcome variable of oral mucositis grade, which is a qualitative ranked variable, was reported as the number and percentage in each group, and the Mann-Whitney test was used to compare the two groups.

The outcome variable of the numerical pain intensity scale is a qualitative variable whose distribution was examined and considered abnormal and median and interquartile range (IQR) were therefore used to describe this variable. The Mann-Whitney test was used to compare the two groups. The level of statistical significance was set at p < 0.05.

## Results

The patients in the intervention group included 29 individuals and the patients in the comparison group included 30 individuals. Demographics, Tumor, and Radiotherapy characteristics of the patients are given in **Table 1**.

**Table 1:** Demographics, Tumor, and Radiotherapy characteristics.

	Intervention group	Comparison group
Age (Mean ± SD)	55.03 ± 9.84	55.57 ± 11.53
Sex (M/F)	17/12	17/13
Type of tumor:		
Nasopharynx	9	5
Buccal SCC	5	9
Lingual SCC	10	12
Other H&N tumors	5	4

**Figure 1:**
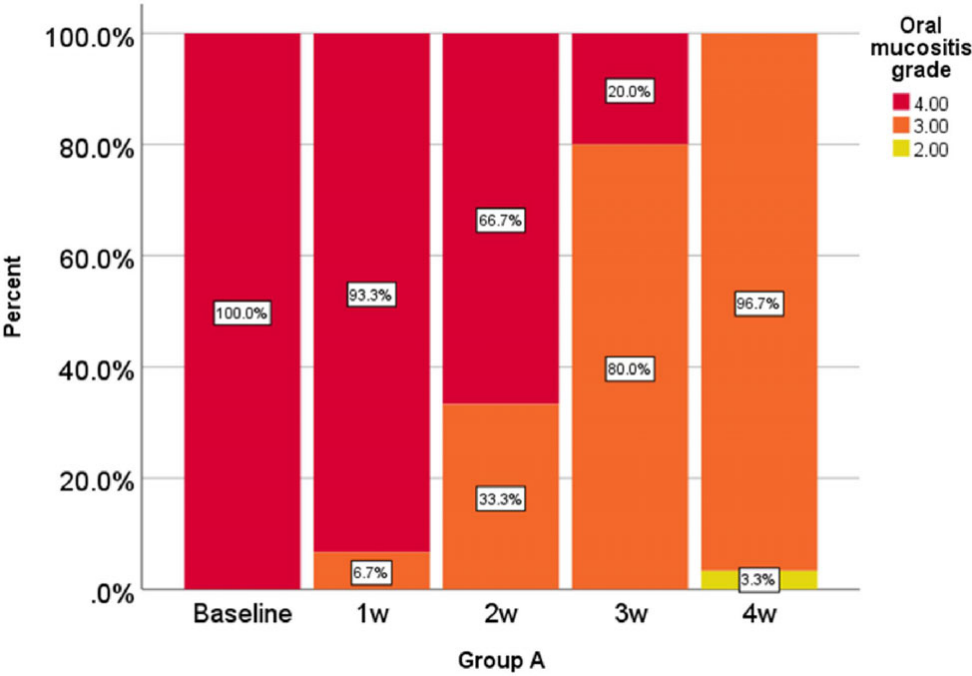
Percentage of oral mucositis grades in the comparison group in weeks 0-1-2-3 and 4.

**Figure 2:**
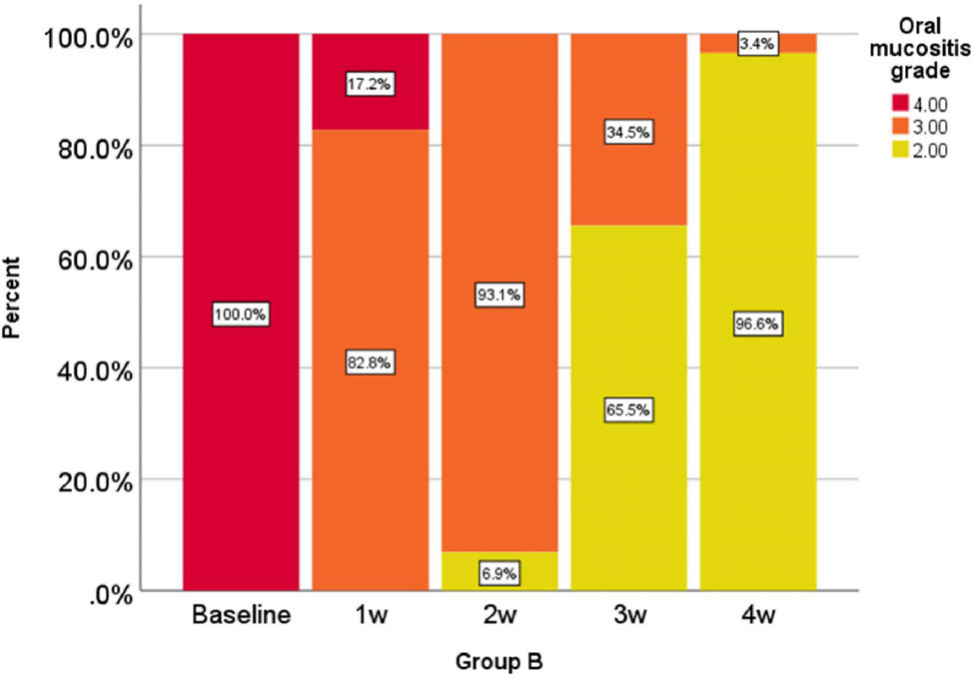
Percentage of oral mucositis grades in the intervention group in weeks 0-1-2-3 and 4.

Oral mucositis grade, represented as a percentage at 0, 1, 2, 3, and 4 weeks in the comparison group is shown in **Figure 1** and oral mucositis grade in the intervention group is given in **Figure 2**.

According to the analysis performed in the first, second, third, and fourth weeks, the reduction of oral mucositis grade in the intervention group was significantly higher than that of the comparison group (P < 0.001).

**Figure 3:**
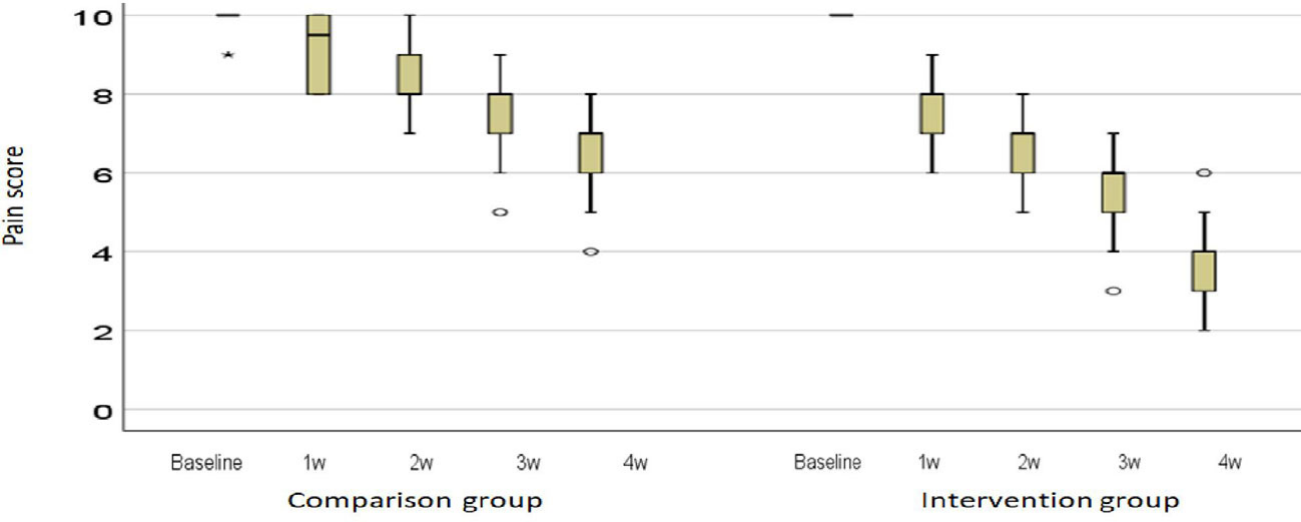
Description of the intensity of pain in the middle and the distance between the quarters. Ο: outlier; *: extreme outlier.

The pain intensity expressed as the median and interquartile range (IQR) is shown in **Figure 3**. In the first, second, third, and fourth weeks, the reduction in pain intensity in the intervention group was significantly higher than in the comparison group (P < 0.001). A photograph of a patient in the intervention group is shown in the first and fourth sessions (Photos 1 and 2).

## Discussion

The results of the present study showed that the combined mouthwash of vitamin E, hyaluronic acid and triamcinolone acetonide could be significantly (P < 0.001) effective on reducing the oral mucositis grade and pain intensity during the first, second, third and fourth weeks of follow-up. One of the side effects of radiation therapy and chemotherapy for head and neck cancers is the occurrence of oral mucositis, which at severe stages, besides increasing medical costs and creating negative psychological and social consequences, causes many limitations such as the prolonged hospitalization time, using liquid diets or total parenteral nutrition (TPN), the increased drug use, and the use of antibacterial, antifungal, and antiviral drugs, which can ultimately lead to the cessation of treatment processes (18). Unfortunately, research conducted from 1980 to 2019 has failed to identify an effective global intervention for the prevention and treatment of oral mucositis. Therefore, oral mucositis treatment remains a medical challenge and requires a standard evidence-based treatment approach (18).

The main bases in the management of radiotherapy-induced oral mucositis are nutritional support, pain alleviation, prophylaxis, and treatment of secondary infections. The previously suggested treatments are locally applied agents (such as Glycyrrhetinic acid/povidone/sodium hyaluronate gel, l-Glutamine, manganese superoxide dismutase, local anesthetics, corticosteroids mouthwashes, and vitamins), systemically applied agents (cyclooxygenase-2 inhibitors, N-acetyl cysteine, transforming growth factor-β3, and systemic corticosteroids), and oral microbial load reduction agents such as antimicrobial and antifungal agents (14).

It is important to recognize and target the pathophysiological processes leading to oral mucositis in order to develop effective preventive and/or therapeutic strategies in this regard. Radiation-induced mucosal damage is actually the result of complex biological and cellular events that occur mainly under the mucosa and eventually lead to epithelial damage (6). Due to the initiating role of reactive oxygen species (ROS) produced during radiation therapy in epithelial cell damage, vitamin E as an antioxidant, and by stabilizing the cell membrane (protecting the cell membrane) against radiation, limit the tissue damage caused by ROS and reduces the severity of oral mucositis during the treatment of head and neck cancers (HNC). Accordingly, that in some studies, it was considered as the main mechanism used to prevent tissue damage (9). In various studies, the desired therapeutic effect of topical vitamin E in oral mucositis has been reported (9, 19). In a meta-analysis in 2017, the significant effect of vitamin E on reducing the severity of oral mucositis in all three groups of patients underwent radiation therapy and/or without chemotherapy and chemotherapy alone was confirmed. This study also showed that the topical form of vitamin E was more effective than its systemic form (8). According to the above-mentioned explanations, in our study, vitamin E was used as one of the components of the combined mouthwash to treat the patients with oral mucositis caused by radiation therapy. However, for the treatment of mucositis, despite its complex pathophysiology, which has been mentioned earlier, the use of a drug with antioxidant properties is not sufficient, and the reason for the temporary effectiveness of single-drug treatments on mucositis is probably due to the failure to observe this point.

Due to the high turnover of the epithelial mucosa, it is exposed to direct damage in chemotherapy and radiotherapy (20). To help repair this mucosal rupture, biological drugs with the ability to stimulate fibroblasts and keratinocytes can be used in topical treatments. In 2001, the U.S. Food and Drug Administration (FDA) approved a gel containing hyaluronic acid as a substance useful in the treatment of oral mucositis and relieving pain (15). Although the exact mechanism associated with the effectiveness of hyaluronic acid in improving the healing process of oral mucositis is not well known, some studies have shown the role of hyaluronic acid compounds only as a physical barrier (coating) between the oral environment and oral mucosa, which reduces pain and possibly improves the healing process (21). Hyaluronic acid (HA) may give rise to re-epithelialization, improve the healing process, and play a role in reducing the size of the erosive/injured areas of the oral mucosa by regulating inflammatory responses and stimulating the proliferation of basal layer keratinocytes (22).

Triamcinolone was chosen as the standard treatment for mucositis, which can be used both as a treatment for the control group and as a base material to be added to vitamin E and hyaluronic acid. Triamcinolone is a kind of fluoride-containing synthetic corticosteroid and an anti-inflammatory compound with medium to high power, which can inhibit all stages of the inflammatory response, from redness and erythema to cell proliferation and wound healing (12). Studies investigating the effects of corticosteroids on mucositis have shown that triamcinolone, as an antioxidant molecule and/or cell protector in the treatment of oral mucositis, can reduce the degree of oral mucositis and the intensity of the pain (12, 20). Furthermore, in previous studies conducted on the treatment of oral mucositis, the compounds of vitamin E, hyaluronic acid and triamcinolone acetonide alone have shown relatively good effects. In the present study, for the first time, the effect of a combined mouthwash containing vitamin E (as an antioxidant), triamcinolone (as an anti-inflammatory agent), and hyaluronic acid (HA) (as a local reducer used for reducing the effects of ROS on the mucosa, with ameliorative effects (improving the healing process) compared to triamcinolone mouthwash alone (comparison group) was investigated in terms of the degree of oral mucositis (according to the WHO criteria) and the severity of pain (according to the VAS criteria). The new composition used substances that have benefits for the treatment of mucositis without the fear of complications of primary tumor growth or increased mortality. For example, studies on the administration of growth factors and cytokines and their side effects are still ongoing (23).

Due to the high molecular weight of vitamin E in combination with the two above-mentioned substances, it was not possible to use the orabase form, which has a longer shelf life in the mouth, and this limitation led to the use of its mouthwash form in this study.

In this study, the WHO classification was used to select patients, which is a combination of objective criteria such as mucosal changes including redness and ulcers and functional criteria such as inability to eat. Studies have shown that this criterion is significantly correlated with the clinical signs and symptoms of mucositis (24).

Due to the lack of a common treatment protocol for the treatment of mucositis, some studies have conducted clinical trials without placing treatment in the control group (25). One of the advantages of the present study was the use of a combination therapy base (triamcinolone) as a control group treatment, which made it possible to compare the effect or non-effect of added substances to the base compared to the base material alone and prevented unreal magnification of the results.

Moreover, previous studies have not reported any significant side effects regarding the use of separate compounds of vitamin E, hyaluronic acid and triamcinolone acetonide in the treatment of oral mucositis, and in the present study, no side effects such as swelling of the lips, tongue, and eyelids, hives or itching in the body were observed in patients either.

According to the results of our study, the use of a combined mouthwash containing vitamin E, hyaluronic acid and triamcinolone acetonide can be an effective treatment in patients with radiotherapy-induced oral mucositis. One of the limitations in the current study was the lack of quality of life’s evaluation. It is suggested to add quality of life’s evaluation through questionnaires into outcome measurements.

In this study, the combined mouthwash of vitamin E, hyaluronic acid and triamcinolone acetonide was found to be effective in the treatment of oral mucositis caused by radiation therapy and positive results were reported in this regard as well, probably due to the antioxidant, anti-inflammatory and improved healing process mechanisms, which are the results of the biological nature of the components of this mouthwash.

## Data Availability Statement

The raw data supporting the conclusions of this article will be made available by the authors, without undue reservation.

## Ethics Statement

The studies involving human participants were reviewed and approved by Tehran University of Medical Sciences. The patients/participants provided their written informed consent to participate in this study.

## Author Contributions

FA-H contributed to conception and design, drafted the manuscript, and gave final approval. MP contributed to acquisition and interpretation, drafted the manuscript, and gave final approval. MA contributed to conception and design, drafted the manuscript, and gave final approval. M-SM contributed to conception and design, contributed to analysis and interpretation, drafted the manuscript, and gave final approval. All authors contributed to the article and approved the submitted version.

## Conflict of Interest

The authors declare that the research was conducted in the absence of any commercial or financial relationships that could be construed as a potential conflict of interest.

## Supplementary Material

Supplementay Datasheet 1 CONSORT diagram.

Supplementary Figure 1 A photograph of a patient (Code 20) in the intervention group in the first sessions. Ulcerative and erosive lesions are seen in the left buccal mucosa, the lips, and the tongue (with Mucositis Grade 4).

Supplementary Figure 2 A photograph of a patient (Code 20) in the intervention group in the fourth sessions (with Mucositis Grade 2).
