# Supplementary material for: Mouthwash Containing Vitamin E, Triamcinolon, and Hyaluronic Acid Compared to Triamcinolone Mouthwash Alone in Patients With Radiotherapy-Induced Oral Mucositis: Randomized Clinical Trial
Source: Front Oncol. 2021 Mar 11;11:614877. doi: 10.3389/fonc.2021.614877 (PMC7991076; doi:10.3389/fonc.2021.614877)
Supplement: Supplementay Datasheet 1 — CONSORT diagram. [file DataSheet_1.doc]

**
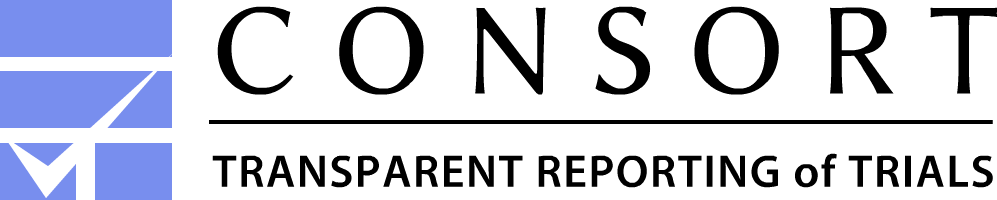
**

**CONSORT 2010 Flow Diagram**

**Allocation**

**Analysis**

**Follow-Up**

**Enrollment**

Assessed for eligibility (n=180)

Excluded (n=120)

♦  Not meeting inclusion criteria (n=110)

♦  Declined to participate (n=10)

♦  Other reasons (n=0)

Analysed (n=29)
♦ Excluded from analysis (give reasons) (n=0)

Lost to follow-up (give reasons) (n=1)

Discontinued intervention (give reasons) (n=0)

Allocated to intervention group (n=30)

♦ Received allocated intervention (n=30)

♦ Did not receive allocated intervention (give reasons) (n=0)

Lost to follow-up (give reasons) (n=0)

Discontinued intervention (give reasons) (n=0)

Allocated to comparison group (n=30)

♦ Received allocated intervention (n=30)

♦ Did not receive allocated intervention (give reasons) (n= )

Analysed (n= 30)
♦ Excluded from analysis (give reasons) (n=0)

Randomized (n=60)
