# Supplementary figures and images for: Mouthwash Containing Vitamin E, Triamcinolon, and Hyaluronic Acid Compared to Triamcinolone Mouthwash Alone in Patients With Radiotherapy-Induced Oral Mucositis: Randomized Clinical Trial
Source: Front Oncol. 2021 Mar 11;11:614877. doi: 10.3389/fonc.2021.614877 (PMC7991076; doi:10.3389/fonc.2021.614877)

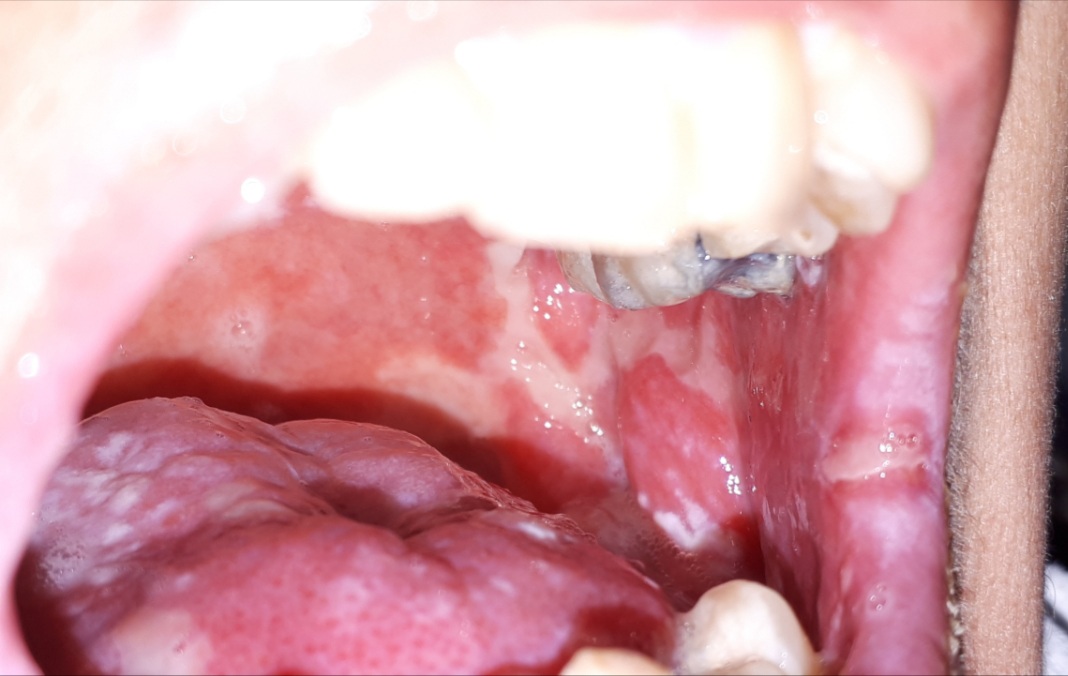

Supplement: Supplementary Figure 1 — A photograph of a patient (Code 20) in the intervention group in the first sessions. Ulcerative and erosive lesions are seen in the left buccal mucosa, the lips, and the tongue (with Mucositis Grade 4). [file Image_1.jpeg]

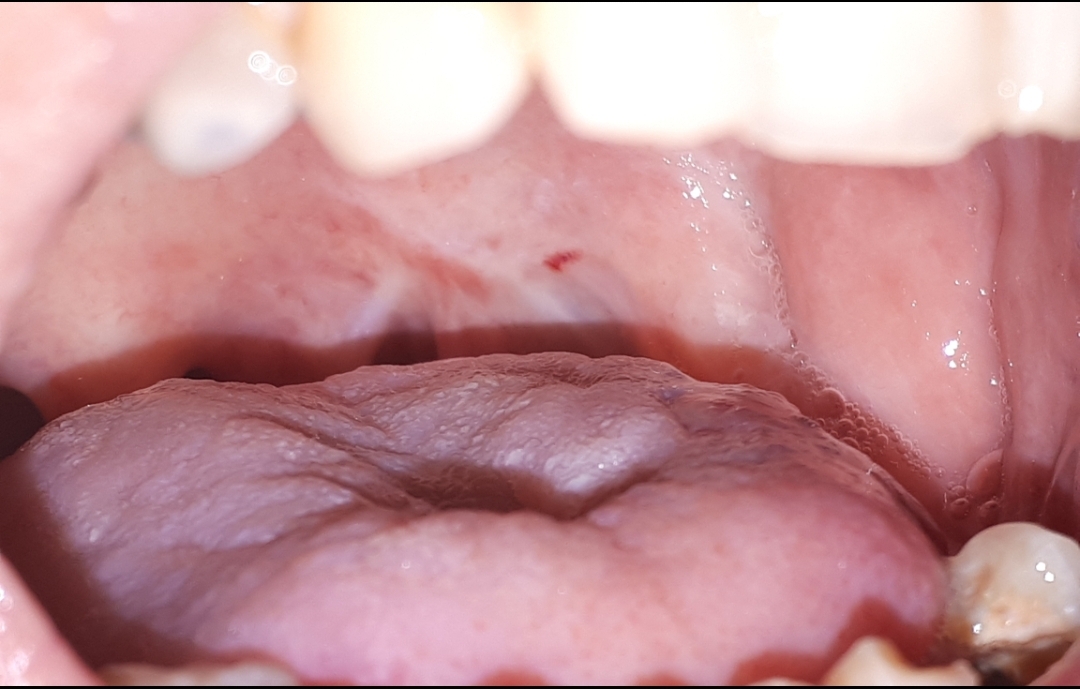

Supplement: Supplementary Figure 2 — A photograph of a patient (Code 20) in the intervention group in the fourth sessions (with Mucositis Grade 2). [file Image_2.jpeg]
